# Enhanced HOXA10 sumoylation inhibits embryo implantation in women with recurrent implantation failure

**DOI:** 10.1038/cddiscovery.2017.57

**Published:** 2017-10-09

**Authors:** Ruiwei Jiang, Lijun Ding, Jianjun Zhou, Chenyang Huang, Qun Zhang, Yue Jiang, Jingyu Liu, Qiang Yan, Xin Zhen, Jianxin Sun, Guijun Yan, Haixiang Sun

**Affiliations:** 1 Reproductive Medicine Center, The Affiliated Drum Tower Hospital of Nanjing University Medical School, Nanjing, People’s Republic of China; 2 Department of Medicine, Center for Translational Medicine, Thomas Jefferson University, Philadelphia, PA, USA

## Abstract

HOXA10 has emerged as an important molecular marker of endometrial receptivity. Recurrent implantation failure (RIF) after *in vitro* fertilization-embryo transplantation (IVF-ET) treatment is associated with impaired endometrial receptivity, but the exact underlying mechanism of this phenomenon remains elusive. Here we found that HOXA10 was modified by small ubiquitin like-modifier 1 (SUMO1) at the evolutionarily conserved lysine 164 residue. Sumoylation inhibited HOXA10 protein stability and transcriptional activity without affecting its subcellular localization. SUMO1-modified HOXA10 expression was decreased in estradiol- and progesterone-treated Ishikawa cells. Sumoylation inhibited the accelerant role of HOXA10 in BeWo spheroid and mouse embryo attachment to Ishikawa cells. Importantly, aberrantly high SUMO1-HOXA10 expression was detected in mid-secretory endometria of women with RIF compared with that of the control fertile women. Together, our results suggest that HOXA10 sumoylation impairs the process of embryo implantation *in vitro* and takes part in the development of RIF.

## Introduction

Embryo implantation is a process of mutual communication between the blastocyst and the uterus primarily under the direction of ovarian estrogen and progestin. These hormones cause the uterus to become receptive to blastocyst implantation 5–8 days after ovulation in a normal human menstrual cycle; this period is called the ‘window of receptivity’.^1^ Several molecules, such as integrins, cytokines and growth modulators, have been found to be associated with the receptive phase,^2^ in which HOXA10 has emerged as an important factor. The *HOXA10* gene belongs to a large family of transcription factors that share a highly conserved homeodomain, which is structurally related to the helix-turn-helix motif of prokaryotic DNA-binding proteins and which has sequence-specific DNA-binding activity. HOXA10 expression in the human uterus is dependent on the menstrual cycle stage, as expression is markedly increased during the mid-secretory phase, corresponding to the time of implantation and increased progesterone levels.^3^ Mice subjected to a targeted disruption of the *HOXA10* gene are infertile because of defective endometrial receptivity.^4^ Decreased HOXA10 expression during the mid-secretory phase has been identified in some gynecological diseases associated with infertility, including endometriosis, adenomyosis and hydrosalpinges.^5–7^ Recently, we reported that aberrantly high p300/CREB-binding protein-associated factor (PCAF) expression results in decreased HOXA10 expression via acetylation in the eutopic endometria of women with endometriosis, which impairs endometrial receptivity and embryo implantation. Endometrial receptivity may be the key factor for women who have high-quality embryos but experience recurrent implantation failure (RIF) after *in vitro* fertilization-embryo transplantation (IVF-ET) treatment. No study has reported HOXA10 expression in patients with RIF, and the molecular mechanisms for the regulation of HOXA10 in implantation failure remain poorly characterized.

The modification of proteins by small ubiquitin like-modifier (SUMO) is an important mechanism for marked post-translational regulation of protein function. Sumoylation involves the covalent attachment of SUMO protein family members to lysine residues in specific target proteins via an enzymatic cascade analogous to but distinct from the ubiquitylation pathway.^8^ Four isoforms of mammalian SUMO have been identified: SUMO1, 2, 3 and 4. SUMO2 and SUMO3 are often referred to as SUMO2/3 because they share 97% identity, whereas SUMO1 shares 50% identity with SUMO2/3. SUMO1 and SUMO2/3 are conjugated to distinct substrates *in vivo* and differ in their ability to form SUMO chains.^9^ SUMOylation can be reversed by SUMO-specific deconjugating enzymes called Ulp/SENPs.^10^ Sumoylation is essential for mammalian development and profoundly modulates the function of many diverse target proteins by altering protein stability, protein–protein interactions, cellular localization and transcriptional activity.^11^ Decidualization is associated with global hyposumoylation and SUMO1 conjugate redistribution into distinct nuclear foci. Decreased levels of SUMO1 modified androgen receptor and SUMO1-modified progesterone receptor A in decidualizing cells account for their increased responsiveness to androgen and progesterone, respectively.^12,13^

In the present study, we demonstrated that HOXA10 ;was modified by SUMO1 and that sumoylation inhibited HOXA10 protein stability and transcriptional activity, leading to impaired attachment rates *in vitro*. Estradiol and progesterone attenuated sumoylated HOXA10, and enhanced amounts of sumoylated HOXA10 were found in the endometria of women who experienced RIF. Our study reveals that the sumoylation status of HOXA10 can have an impact on the process of embryo implantation and may contribute to the development of RIF.

## Results

### SUMO1 modifies HOXA10 on its evolutionarily conserved lysine 164 residue

**Figure 1:**
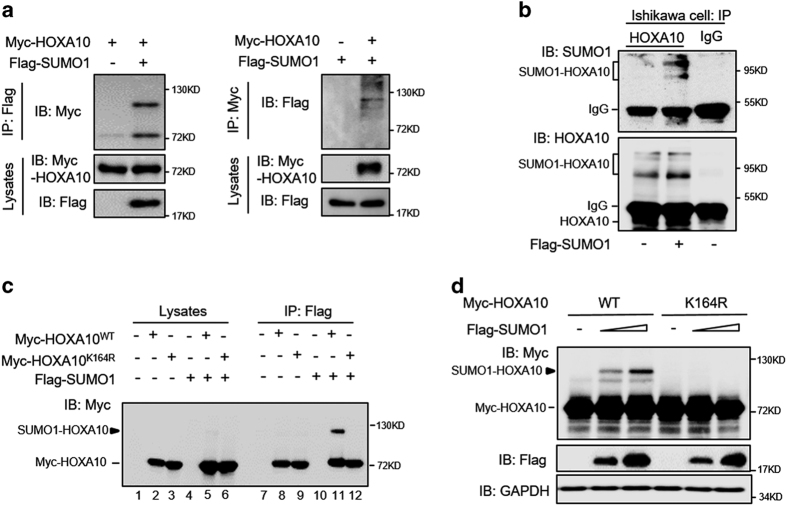
SUMO1 modifies HOXA10 on its evolutionarily conserved lysine 164 residue. (**a**) HEK293T cells were co-transfected with Myc-HOXA10 and Flag-SUMO1. After 48 h, the cell lysates were affinity-purified using anti-Flag-agarose and anti-Myc-agarose, respectively, and then blotted with anti-Myc and anti-Flag antibodies. An increased molecular weight of ~30 kDA of the detected HOXA10 was observed when HOXA10 and SUMO1 were coexpressed. (**b**) Extracts from Ishikawa cells transfected with Flag-SUMO1 or an empty vector were incubated with the anti-HOXA10 antibody, and then blotted with anti-HOXA10 and anti-SUMO1 antibodies. Goat IgG was used as a negative control for the co-IP procedure. (**c**) HEK293T cells were co-transfected with the indicated plasmid. After 48 h, the cell lysates were subjected to anti-flag-agarose precipitation and then immunoblotted with the anti-Myc antibody. (**d**) HEK293T cells were co-transfected with the indicated plasmids. After 48 h, the cell lysates were directly subjected to western blot.

Immunoprecipitation analysis of the HEK293T cell extracts showed that the detected HOXA10 had an increased molecular weight of ~30 kDa only when HOXA10 and SUMO1 were coexpressed (Figure 1a). Endogenous HOXA10 with an increased molecular weight of ~30 kDa was recognizable by both anti-SUMO1 and anti-HOXA10 antibodies in Ishikawa cells with SUMO1 overexpression (Figure 1b). *In silico* analyses of HOXA10 using SUMOplot (Abgent) and SUMOsp 2.0 prediction analysis programs pointed to lysine 164 as the only amino-acid residue modified in HOXA10.^14^ The critical residues of the IKEE motif are conserved in vertebrate HOXA10 proteins, supporting a functional role for this motif (Supplementary Figure S1A). The SUMO1-HOXA10 observed in cells transfected with wild-type HOXA10 (HOXA1^WT^) was not observed in cells transfected with the K164R mutant in the presence of SUMO1 (Figure 1c, compare lanes 11 and 12). Transfection of Myc-HOXA10^WT^ or Myc-HOXA10^K164R^ alone resulted in the pull down of significant amounts of HOXA10^WT^ and HOXA10^K164R^ proteins, respectively (Figure 1c, lanes 8 and 9), likely due to interactions between the Flag peptide and a special HOXA10 protein motif.^15^ Increased SUMO1 expression resulted in increased SUMO1-HOXA10^WT^ expression, but no changes were observed regarding HOXA10^K164R^ expression (Figure 1d), further proving that K164 is the major sumoylation site of HOXA10. In addition, we also found an interaction between HOXA10 and SUMO2 (Supplementary Figure S1B).

### SUMO1 site disruption by K164R does not alter HOXA10 localization patterns

**Figure 2:**
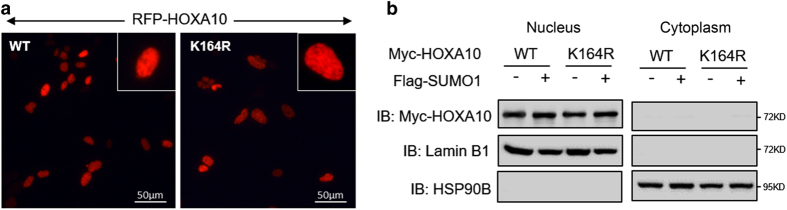
SUMO1 site disruption by K164R does not alter HOXA10 localization. (**a**) Fluorescence staining of HEK293T cells transfected with RFP-HOXA10^WT^ and RFP-HOXA10^K164R^. HOXA10 is shown in red. Bar=50 *μ*m. (**b**) Total cytoplasmic and nucleus proteins were isolated using a nuclear and cytoplasmic protein extraction kit, and the levels of HOXA10^WT^ and HOXA10^K164R^ were determined by western blotting.

SUMO conjugation alters the intranuclear localization of some transcription factors. Fluorescence image analysis showed that HOXA10 mutants were predominantly localized in the nucleus and were indistinguishable from HOXA10^WT^ (Figure 2a). Furthermore, SUMO1 overexpression did not alter the subcellular localization of HOXA10^WT^ or HOXA10^K164R^ (Figure 2b).

### Sumoylation inhibits HOXA10 protein stability and transcriptional activity

**Figure 3:**
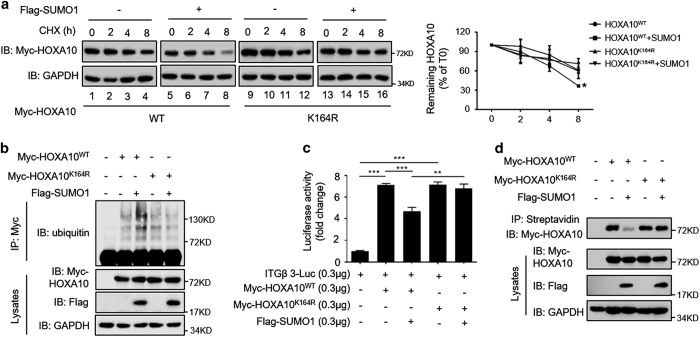
Sumoylation inhibits HOXA10 protein stability and transcriptional activity. (**a**) Ishikawa cells were transfected with Myc-HOXA10^WT^ or Myc-HOXA10^K164R^ and Flag-SUMO1. After 48 h, 50 *μ*g/ml CHX was added to the cells for 2, 4 and 8 h. The cell extracts were subjected to western blot analysis. The levels of remaining HOXA10 were normalized to GAPDH and plotted relative to the levels at the 0-h time point. Values are expressed as the means±S.E.M. (*n*=3), *P<0.05 *versus* HOXA10^WT^ by itself. (**b**) For detection of ubiquitination. Ishikawa cells were treated with 10 *μ*M MG132 for 6 h before being harvested and subjected to immunoprecipitation with anti-Myc antibody, followed by western blot analysis to assess protein expression. (**c**) Ishikawa cells were transfected with ITGB3-Luc, Myc-HOXA10^WT^, Myc-HOXA10^K164R^ and Flag-SUMO1 as indicated. After 48 h, the luciferase activities were measured, and the activity levels are presented as the fold induction. The error bars indicate±S.E.M. of three independent experiments. **P<0.01; ***P<0.001. (**d**) For the biotin-labeled DNA pull-down assay, Ishikawa cell extracts were incubated with biotinylated DNA probe containing HOXA10-binding sites (TTAT). After the cell extracts were incubated with streptavidin Sepharose beads, the extracts were subjected to western blot analysis.

Similar to the effects of post-translational modification by ubiquitin, SUMO modification regulates protein degradation and stability. When *de novo* protein synthesis was blocked by cycloheximide (CHX), we observed that HOXA10^WT^ and HOXA10^K164R^ had equal protein stability in the absence of SUMO1 (Figure 3a, compare lanes 1–4 with lanes 9–12). In the presence of SUMO1, HOXA10^WT^ was degraded more rapidly than in HOXA10^WT^-expressing cells (Figure 3a, compare lanes 1–4 with lanes 5–8), whereas no change in HOXA10K^164R^ was found, suggesting that SUMO1 modifies K164 of HOXA10 and inhibits its protein stability. We observed similar levels of ubiquitination between HOXA10^WT^ and HOXA10^K164R^, whereas overexpression of SUMO1 markedly increased the ubiquitination of HOXA10, indicating that sumoylation somehow promotes ubiquitin-mediated degradation (Figure 3b). HOXA10K^164R^ and HOXA10^WT^ displayed similar abilities to activate the reporter reporter plasmid of *β*3-integrin (ITGB3-Luc), and overexpression of SUMO1 alone did not significantly inhibit ITGB3-luc reporter gene activity (Supplementary Figure S3A). HOXA10 and SUMO1 coexpression significantly inhibited HOXA10-mediated increased reporter gene activity by 34% (*P*<0.001), although no significant difference in HOXA10^K164R^ was found (Figure 3c). A DNA pull-down assay with the HOXA10 DNA-binding consensus sequence showed that sumoylation severely compromised the ability of HOXA10 to bind the response element (Figure 3d), suggesting that sumoylation diminishes HOXA10-mediated transactivation by decreasing its DNA-binding capacity. PCAF has been proven to downregulate HOXA10 protein stability and transcriptional activity by acetylating K338 and K339.^16^ In this study, abolishing the K164 sumoylation site promoted the acetylation of HOXA10 compared with that of HOXA10^wt^, suggesting an interaction between sumoylation and acetylation in the modification of HOXA10 (Supplementary Figure S2B).

### Estradiol and progesterone inhibit HOXA10 sumoylation

**Figure 4:**
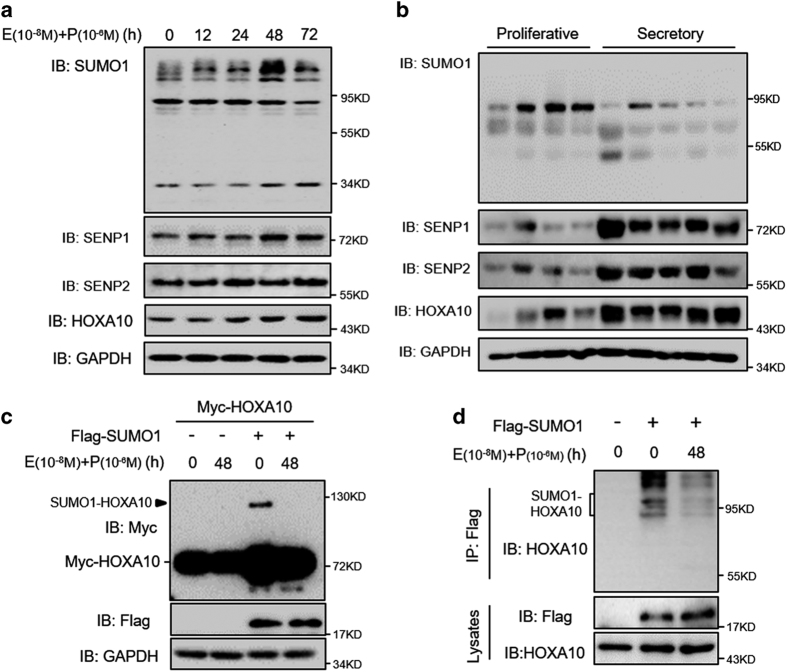
Estradiol and progesterone inhibit HOXA10 sumoylation. (**a**) Ishikawa cells were treated with estradiol (E; 10^−8^ M) and progesterone (P; 10^−6^ M) for various amounts of time as indicated. Whole-cell lysates were analyzed by western blot with the indicated antibodies. (**b**) Proliferative (*n*=4) and secretory (*n*=5) endometria were obtained from normal women, and protein lysates were analyzed by western blot with the indicated antibodies. (**c**) Ishikawa cells transfected with Myc-HOXA10 alone or together with Flag-SUMO1 were treated with estradiol (E; 10^−8^ M) and progesterone (P; 10^−6^ M) for 48 h. Whole-cell lysates were analyzed by western blot. (**d**) Ishikawa cells either transfected with Flag-SUMO1 or not were treated with estradiol (E; 10^−8^ M) and progesterone (P; 10^−6^ M) for 48 h. Whole-cell lysates were affinity-purified using anti-Flag-agarose and then analyzed by western blot.

Ishikawa cells express estrogen and progestin receptors and serve as an endometrial epithelium model.^17^ We found that HOXA10 expression increased over time in Ishikawa cells treated with estradiol and progesterone, but that expression of SUMO1 conjugates with a molecular weight of 90 kDa decreased over time (Figure 4a). However, no obvious changes in SUMO2/3-conjugate expression were found (Supplementary Figure S2C). In addition, SUMO-specific deconjugating enzymes, SENP1 and SENP2, increased over time. Similarly, decreased SUMO1 conjugates and increased SENP1 and SENP2 expression were detected in secretory endometria compared with that of proliferative endometria (Figure 4b). Interestingly, there was an increased expression of SUMO2 conjugates in secretory endometria compared with that of proliferative endometria (Supplementary Figure S2D). We found markedly attenuated SUMO1-HOXA10 expression in Ishikawa cells treated with estradiol and progesterone (Figure 4c). Furthermore, endogenous HOXA10 sumoylation was obviously diminished in cells treated with estradiol and progesterone (Figure 4d). These results suggest that estradiol and progesterone inhibit HOXA10 sumoylation.

### Sumoylation inhibits HOXA10-promoted embryo attachment *in vitro*

**Figure 5:**
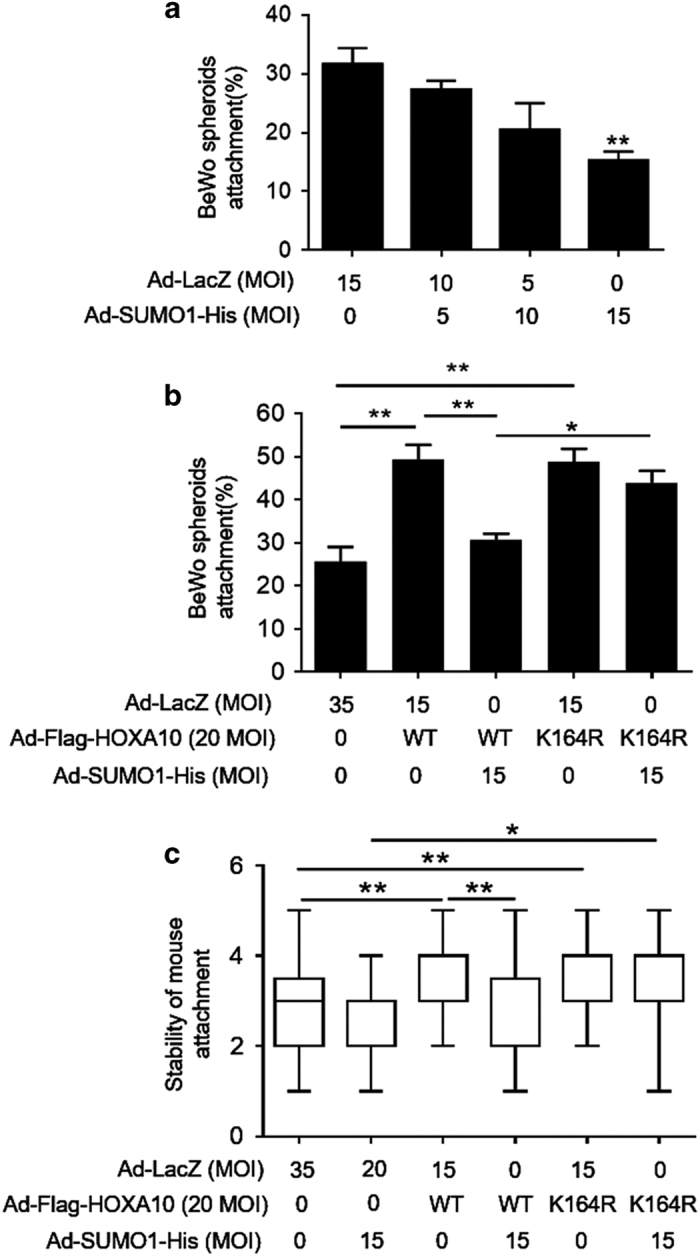
Sumoylation inhibits HOXA10-promoted embryo attachment *in vitro*. (**a** and **b**) Adhesion experiments with BeWo spheroids attached to the Ishikawa cell monolayer. The data are the average of three independent experiments (*n*=3). The error bars indicate ± S.E.M. of three independent experiments. An ANOVA test was used to compare the percentage of attached spheroids with each treatment in comparison with that of the control. (**c**) Adhesion experiments with mouse embryos attached to the Ishikawa cell monolayer. The results are expressed as the median with the interquartile range of three independent experiments; boxes indicate quartiles and whiskers indicate range. Kruskal–Wallis with Dunn’s multiple comparison tests were used among these groups. *P<0.05; **P<0.01.

To determine the functionality of HOXA10 sumoylation in embryo attachment, we first used a relevant *in vitro* model of a confluent monolayer of Ishikawa cells co-cultured with BeWo spheroids. The attachment experiments with BeWo spheroids demonstrated that SUMO1 overexpression decreased BeWo spheroid attachment in a dose-dependent manner (Figure 5a). HOXA10 and SUMO1 coexpression significantly decreased BeWo spheroid attachment compared with that of HOXA10 alone (*P*<0.05), but SUMO1 overexpression did not impair HOXA10^K164R^-promoted BeWo spheroid attachment significantly (Figure 5b). To further confirm the negative effect of HOXA10 sumoylation on embryo attachment, an assay involving an *in vitro* model of mouse embryo attachment was used. Embryos incubated with HOXA10^WT^-overexpressing Ishikawa cells had the attachment score at 4 (3–4); median (interquartile range)), but HOXA10^WT^ and SUMO1 coexpression group got a significantly decreased attachment score (2 (2–3.5]), *P*<0.01). SUMO1 overexpression did not inhibit HOXA10^K164R^-promoted mouse embryo attachment significantly (Figure 5c). These data suggest that sumoylation inhibits HOXA10-promoted embryo attachment.

### Women with RIF display aberrantly high SUMO1-HOXA10 expression

**Figure 6:**
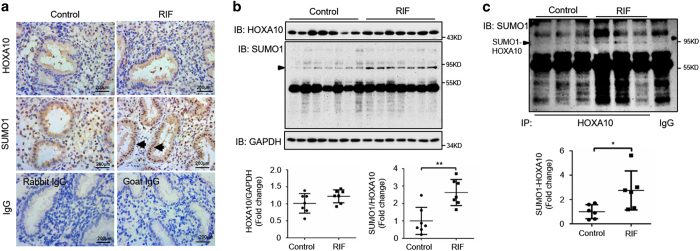
Aberrantly high HOXA10-SUMO1 expression in the endometria of women with RIF. (**a**) Timed mid-secretory endometrial biopsies from normal control women and infertile women with RIF were analyzed using IHC. Goat IgG and rabbit IgG were used as negative controls. Arrows show increased SUMO1 conjugates in glandular epithelia. (**b**) Timed mid-secretory endometrial biopsies from normal control women (*n*=7) and infertile women with RIF (*n*=7) were analyzed for HOXA10 and SUMO1 expression using western blot. (**c**) Timed mid-secretory endometrial biopsies from healthy control women (*n*=6) and infertile women with RIF (*n*=6) were analyzed. Tissue proteins were affinity-purified using the anti-HOXA10 antibody. The goat IgG antibody was used as a negative control. Protein levels were normalized to the GAPDH protein expression levels. Student’s *t*-tests were used for comparisons of two groups. *P<0.05; **P<0.01.

We next evaluated the role of HOXA10 sumoylation in RIF. Immunohistochemistry assays showed that there were no significant differences in the expression and localization of HOXA10 between control fertile women and women with RIF, whereas the expression of SUMO1 conjugates was increased in women with RIF, especially in their glandular epithelia (Figure 6a). Western blot analysis showed similar levels of HOXA10 expression in the control fertile women and women with RIF, whereas women with RIF presented significantly higher levels of SUMO1 conjugates with a molecule weight of ~90 kDa (*P*<0.05; Figure 6b). In addition, no changes in the expression of the level of SUMO2/3 conjugates were observed (Supplementary Figure S3C). Aberrantly higher levels of SUMO1-HOXA10 were found in the endometria of women with RIF compared with those of the control fertile women (*P*<0.05; Figure 6c), suggesting that HOXA10 sumoylation may contribute to the development of impaired endometrial receptivity and embryo implantation in RIF.

## Discussion

In this study, we showed that HOXA10 is post-translationally and covalently modified by SUMO1 conjugation on lysine 164. Sumoylation-deficient HOXA10^K164R^ achieved greater protein stability and transcriptional activity compared with that of HOXA10^WT^ in the presence of SUMO1. Estradiol and progesterone inhibit HOXA10 sumoylation, which is the most important factor of endometrial receptivity and embryo implantation regulation. In contrast, aberrant conjugation of SUMO1 impairs HOXA10-promoted embryo implantation, which may contribute to RIF.

SUMO conjugation often requires the ψKXE sequence, in which ψ is a large hydrophobic amino acid and X represents any amino acid.^18^ The classic strategy for studying protein sumoylation is mutating the specific lysine residue believed to be the sumoylation site. The conservative K-to-R mutation is usually used because of the great similarity between these two amino acids compared with that of other amino acids.^19^ In addition to sumoylation, several other protein modifications, such as methylation, acetylation and ubiquitination, occur on lysine residues. Our previous research demonstrated that PCAF decreases the transactivation function of HOXA10 dependent on its histone acetyltransferase activity and acetylates HOXA10 at lysines 338 and 339, which are both located in the homeodomain.^16^ The activity of HOX proteins largely depends on their ability to control gene expression as DNA-binding proteins through the homeodomain, which is the defining motif of this family.^20^ HOXA10 was modified with SUMO1 at K164 in the N-terminal region, and little is known about the function of this region. Some studies have indicated that the N-terminal region of HOXA10 is required for the rib-repressing activity of HOXA10 in mouse development.^21^ Some studies have proven the assembly of poly-SUMO1 chain on some protein substrate, including TOP1^22^ and EV71 3C.^23^ There were several slower migrating bands found in endogenous HOXA10, most probably because of the formation of poly-SUMO1 chain. However, there was only one slower migrating band in exogenous HOXA10, maybe due to the difference between endogenous and exogenous HOXA10. We found that sumoylation inhibits the transcriptional activity and DNA-binding capacity of HOXA10 and interacts with its ubiquitination and acetylation. Our study contributes to further understanding the functions of various regions of HOXA10.

Sumoylation regulates the intracellular localization of several proteins, including cellular retinoic acid-binding protein II (CRABP-II), Daxx, NEMO and SMAD4.^24–27^ HOXA10 is synthesized in the cytosol and then transported into the nucleus, where it functions as a transcription factor. Protein transport from the cytosol into the nucleus is mediated by a nuclear localization signal (NLS), which binds to a heterodimeric receptor on the membrane.^28^ A computer program (www.predictprotein.org) predicted that the HOXA10 homeodomain contains a putative NLS sequence. Our data demonstrated that sumoylation is not involved in the regulation of intracellular HOXA10 localization.

Successful implantation requires a receptive endometrium, a functional embryo at the blastocyst developmental stage and a synchronized dialogue between maternal and embryonic tissues.^29^ The endometrium becomes receptive under the stimulation of estrogen and progestin during the ‘window of implantation’.^30^ Both estrogen and progestin act independently and in concert to upregulate HOXA10 expression in the endometrium.^3^ In turn, HOXA10 regulates endometrium receptivity and decidualization by the activation or repression of a number of downstream markers specific to the implantation window, including *β*3 integrin and insulin-like growth factor-binding protein-1.^31,32^ The importance of maternal HOXA10 expression in implantation has been proven in HOXA10-knockout mice and mice injected with antisense oligonucleotides of HOXA10.^33^ In our study, increased HOXA10 expression was correlated with increased estrogen and progestin, but SUMO1-conjugated proteins showed a contrary tendency *in vitro* and *in vivo*. The co-immunoprecipitation assay determined that estradiol and progesterone treatment attenuated the sumoylation of HOXA10.

Assisted reproductive technology tools are now available that enable the selection of high-quality embryos, but implantation rates are still relatively low and have not increased sufficiently in the past decade to allow the widespread adoption of single-embryo transfer.^34^ A functioning and receptive endometrium is crucial for embryo implantation. HOXA10 expression is decreased during the secretory phase in subfertile women who have endometriosis, hydrosalpinx, leiomyoma and PCOS.^7^ Although no changes in HOXA10 protein expression were found in the endometria of women with RIF, sumoylated HOXA10 expression was aberrantly higher in these patients compared with that of fertile control women, suggesting that HOXA10 sumoylation may contribute to impaired endometrial receptivity and embryo implantation failure. Hyposumoylation of HOXA10 in response to estradiol and progesterone treatment occurred, as SUMO1 conjugation was generally decreased in normal secretory endometria compared with that of normal proliferative endometria, suggesting that decreased SUMO1 conjugation may be necessary for normal embryo implantation. This marked change in global sumoylation may be a result of increased SENP1/2 regulating reversible sumoylation,^35^ providing new insight into endometrial receptivity.

In summary, our study provides the first evidence that HOXA10 is post-translationally modified by SUMO1 conjugation on lysine 164, which is under the regulation of estradiol and progesterone. Enhanced SUMO1-HOXA10 inhibits embryo attachment, which may be a potential cause of RIF. However, global sumoylation in response to hormone stimulation and impaired embryo implantation is not restricted to HOXA10, and further studies are required to investigate the function of sumoylation in endometrial receptivity and embryo implantation.

## Materials and methods

### Patients and sample collection

Endometrial biopsies for this study were obtained from women attending the Center for Reproductive Medicine of Nanjing Drum Tower Hospital. All samples were collected with the informed consent of the patients, and approval from the ethics committee was obtained for this study. Secretory endometria were obtained from seven normal women and seven women with RIF after IVF-ET. Proliferative endometria were obtained from four normal women. The normal group was composed of women whose infertility was due to male factors and who were confirmed to be fertile after their first IVF-ET treatment. RIF is defined as the absence of implantation after two consecutive cycles of IVF, intracytoplasmic sperm injection or frozen embryo replacement cycles in which the cumulative number of transferred embryos was no less than four for cleavage-stage embryos and no less than two for blastocysts, with all embryos being of good quality.^36^ All women had a good hormonal reserve (follicle stimulating hormone on day 3 of the cycle<10 mIU/ml).

### Cell culture and treatment

Ishikawa and BeWo cells were maintained in DMEM/F12 supplemented with 10% fetal bovine serum (Gibco BRL/Invitrogen, Carlsbad, CA, USA) and 1% penicillin/streptomycin (HyClone, South Logan, UT, USA). For treatment with estradiol and progesterone, cells were cultured in Phenol red-free DMEM/F12 supplemented with 10% charcoal-stripped fetal bovine serum for 24 h before being treated with 10^−8^M 17 *β*-estradiol (Sigma, St. Louis, MO, USA) and 10^−6^ M progesterone (Sigma) for various durations. For ubiquitination detection, cells were treated with 10 *μ*M MG132 (Sigma) for 6 h before being harvested. CHX (Sigma) was added for the indicated times at a final concentration of 50 *μ*g/ml for the protein degradation assay.

### Construction of plasmid and adenovirus vectors

The pCS2-Myc-HOXA10 expression plasmid and adenovirus vector harboring the full-length HOXA10 gene (Ad-Flag-HOXA10) was previously constructed.^16^ Lysine to arginine mutant of HOXA10 K164R was generated by using the Quickchange site-directed mutagenesis kit (Stratagene, La Jolla, CA, USA). The Flag-SUMO1 expression plasmid (Plasmid #49046) was purchased from Addgene (Cambridge, MA, USA). SUMO1-His Adenovirus (136904A) was purchased from Abm (Richmond, BC, Canada). The adenovirus bearing LacZ (Ad-LacZ) was obtained from Clontech (Palo Alto, CA, USA) and was used as a control in the adenovirus-mediated HOXA10 and SUMO1 overexpression experiments. The virus was packaged and amplified in HEK293A cells and purified using CsCl banding.

### DNA pull-down assay

The biotin-labeled DNA probes were synthesized by Sangon Biotech (Shanghai, China). The triple sequence repeats of HOXA10-binding sites (TTAT) contained biotin-3xTTAT-F (Biotin-5′- TCGATATTTATTCTTTTGAATTATGCTTATATCTTTT-3′) and 3xTTAT-R (5′- AAAAGATATAAGCATAATTCAAAAGAATAAATATCGA-3′). ssDNA was thermally annealed to form dsDNA before performing the pull-down experiments. Cell lysates were mixed with biotinylated DNA probe and incubated at 4 °C overnight. Streptavidin Sepharose beads were then added, and the mixture was incubated for 4 h at 4 °C. The beads were washed three times with 1× PBS and resolved by 2× SDS loading buffer for western blot analysis.

### Western blot analysis

Tissues and cells were homogenized in whole-cell lysis buffer (50 mM Tris-HCl (pH 7.6), 150 mM NaCl and 1.0% NP-40) containing a protease inhibitor cocktail and 20 mM N-ethylmaleimide to prevent SUMO de-conjugation. Immunoblotting was performed with primary antibodies against HOXA10 (1 : 1000; Santa Cruz, Santa Cruz, CA, USA), SUMO1 (1 : 1000; Abcam, Cambridge, CA, USA), SENP1 (1 : 1000; Abcam), SENP2 (1 : 1000; Abgent, San Diego, CA, USA), Flag (1 : 1000; Sigma), Myc (1 : 5000; Invitrogen, Carlsbad, CA, USA), Lamin B1 (1 : 1000; Bioworld, St Louis Park, MN, USA), HSP90B (1 : 1000; Bioworld), ubiquitin (1 : 1000, Abcam), acetylated lysine (1 : 1000; CST, Danvers, MA, USA) and GAPDH (1 : 10 000; Bioworld), followed by donkey anti-goat or goat anti-rabbit secondary antibody conjugated with HRP. Detection was performed using an enhanced chemiluminescence kit (Millipore, Billerica, USA).

### Immunohistochemistry

Endometrial tissues were fixed in 10% neutral-buffered formalin for 24 h, routinely processed and embedded in paraffin. Tissue sections were immunostained with primary antibodies against HOXA10 (1 : 50; Abcam), SUMO1 (1 : 100; Abcam), SUMO2/3 (1 : 100, Abgent) overnight at 4 °C, followed by incubation with rabbit anti-goat IgG or goat anti-rabbit IgG and an avidin-biotin complex (Boster, Wuhan, China) for 1 h each at room temperature. Finally, the sections were stained with 3,3′-diaminobenzidine and counterstained with hematoxylin. Control sections were run concurrently with the experimental sections using nonspecific goat IgG and rabbit IgG, and they were similarly pretreated. Nonspecific staining was not detected in the controls.

### Fluorescence assay

HEK293T cells grown on 8-well microscope slides (Millipore) were transfected with RFP-HOXA10WT or RFP-HOXA10K164R expression plasmid. After 48 h, cells were fixed with 4% paraformaldehyde in PBS (vol/vol) for 20 min at room temperature. After washing with PBS, the slides were covered with coverslips. Digital images were captured using a Leica DM 3000 fluorescence microscope and LAS Core software (Leica Microsystems Limited, Wetzlar, Germany).

### Luciferase reporter assay

The pGL3-basic luciferase reporter plasmid loaded with the ITGB3 promoter was described previously.^16^ Pre-confluent (60%) Ishikawa cells in 12-well plates were transfected with the indicated plasmids. Cells were harvested, and the luciferase activities were analyzed after 48 h using the Dual-Luciferase Assay System (Promega, Madison, WI, USA). Luciferase activity was measured using a luminescence counter (Berthold Technologies, KG, Germany) according to the manufacturer’s instructions. Firefly luciferase activity was normalized for transfection efficiency to the corresponding *Renilla* luciferase activity.

### Attachment assay for BeWo spheroid and mouse embryo attachment to Ishikawa cells

According to our standard laboratory protocol, multicellular spheroids of human choriocarcinoma BeWo cells and mouse blastocysts with endometrial Ishikawa cells were used as *in vitro* models of attachment.^37^ Briefly, a single-cell suspension of BeWo cells was transferred to a Petri dish coated with the anti-adhesive polymer poly-2-hydroxyethyl methacrylate (Sigma) to induce the formation of BeWo spheroids that were 150–200 *μ*m in diameter. Simultaneously, the confluent monolayer of Ishikawa cells was infected with the indicated adenovirus vectors in a 24-well culture plate for 48 h. Fifty spheroids were transferred per chamber onto the confluent monolayer of Ishikawa cells. After the spheroids were incubated for 1.5 h, the attached spheroids were counted, and the attachment rate was expressed as a percentage of the total number of spheroids (% adhesion). Similarly, collected mouse blastocysts were transferred onto Ishikawa cells infected with the indicated adenovirus vectors in a 24-well culture plate, with 10 blastocysts per well. After 24 h, a standardized plate movement protocol was implemented to measure the embryo attachment stability.

### Statistical analysis

Each experiment was repeated at least three times. All values are expressed as the means±S.E.M. or median and interquartile range, as specified. Student’s *t*-tests were used for comparisons of two groups, ANOVA was applied for experiments involving more than two groups and Kruskal–Wallis with Dunn’s multiple comparison tests were used to test the stability of embryo attachment to Ishikawa cells. A *P*-value<0.05 was considered statistically significant.

## Additional information

**Publisher’s note:** Springer Nature remains neutral with regard to jurisdictional claims in published maps and institutional affiliations.
