# Supplementary material for: Enhanced HOXA10 sumoylation inhibits embryo implantation in women with recurrent implantation failure
Source: Cell Death Discov. 2017 Oct 9;3:17057–. doi: 10.1038/cddiscovery.2017.57 (PMC5632741; doi:10.1038/cddiscovery.2017.57)
Supplement: Supplementary Figure Legends [file cddiscovery201757-s1.docx]

Figure S1. (A) Schematic diagram of the evolutionary conserved SUMO1 motif of HOXA10. HOXA10 is modified by SUMO1at lysine 164 site in different species. (B) HOXA10 is covalently modified by SUMO2. HEK293T cells were co-transfected with Myc-HOXA10 and Flag-SUMO2. After 48 hours, the cell lysates were affinity purified using anti-Flag-agarose and anti-Myc-agarose respectively, and then blotted with anti-Myc and anti-Flag antibodies. An increased molecular weight of ~30 kDA of the detected HOXA10 was observed when HOXA10 and SUMO2 were co-expressed.

Figure S2. (A) HOXA10^K164R^ showed similar transcriptional activity to HOXA10^WT^. Ishikawa cells were transfected with indicated plasmid vectors. After 48 hours, the luciferase activities were measured, and the activity levels are presented as the fold induction. The error bars indicate ±SEM of 3 independent experiments. *, P<0.05. (B) Interaction between sumoylation and acetylation in the modification of HOXA10. Ishikawa cells were co-transfected with Myc-HOXA10^WT^, Myc-HOXA10^K164R^ and Flag-SUMO1 as indicated, then subjected to Western blot with anti-AcK antibody. (C) Ishikawa cells were treated with estradiol (E) (10^-8^ M) and progesterone (P) (10^-6^ M) for various amounts of time as indicated. Whole-cell lysates were analysed by Western blot with anti-SUMO2/3 antibody. (D) Proliferative (n=4) and secretory (n=5) endometria were obtained from normal women, and protein lysates were analysed by Western blot with anti-SUMO2/3 antibody.

Figure S3. (A) Ishikawa cells transfected with Flag-HOXA10 expressing adenovirus (Ad-Flag-HOXA10) and SUMO1-His expressing adenovirus (Ad-SUMO1-His) were subjected to Western blot with the indicated antibodies. (B) Phase contrast images captured in upper or lower planes of mouse embryos attached to Ishikawa cells showing the stages of embryo attachment. Images were captured at a magnification of 20×. (C) Timed mid-secretory endometrial biopsies from normal control women and infertile women with RIF were analysed using IHC and Western Blot.
