# Supplementary material for: Enhanced HOXA10 sumoylation inhibits embryo implantation in women with recurrent implantation failure
Source: Cell Death Discov. 2017 Oct 9;3:17057–. doi: 10.1038/cddiscovery.2017.57 (PMC5632741; doi:10.1038/cddiscovery.2017.57)
Supplement: Supplementary Figures [file cddiscovery201757-s2.pdf]

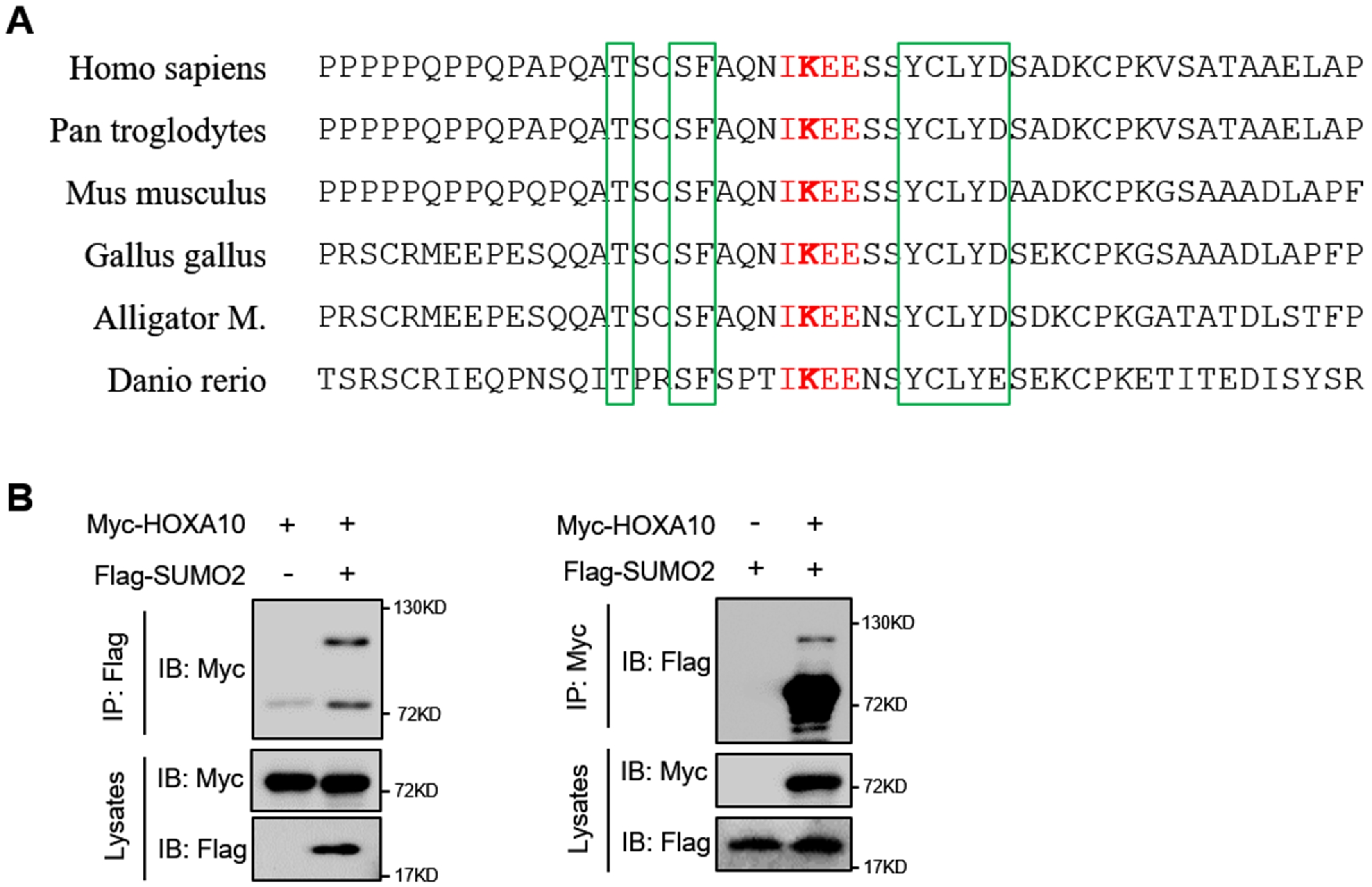
A
Homo sapiens PPPPPQPPQPAPQATSCSFAQNIKEESSYCLYDSADKCPKVSATAAELAP
Pan troglodytes PPPPPQPPQPAPQATSCSFAQNIKEESSYCLYDSADKCPKVSATAAELAP
Mus musculus PPPPPQPPQPQPQATSCSFAQNIKEESSYCLYDAADKCPKGSAAADLAPF
Gallus gallus PRSCRMEEPESQQATSCSFAQNIKEESSYCLYDSEKCPKGSAAADLAPFP
Alligator M. PRSCRMEEPESQQATSCSFAQNIKEENSYCLYDSDKCPKGATATDLSTFP
Danio rerio TSRSCRIEQPNSQITPRSFSPTIKEENSYCLYESEKCPKETITEDISYSR
B
Myc-HOXA10 + +
Flag-SUMO2 - +
IP: Flag
IB: Myc
Lysates
IB: Myc
IB: Flag
130KD
72KD
72KD
17KD
Myc-HOXA10 - +
Flag-SUMO2 + +
IP: Myc
IB: Flag
Lysates
IB: Myc
IB: Flag
130KD
72KD
72KD
17KD

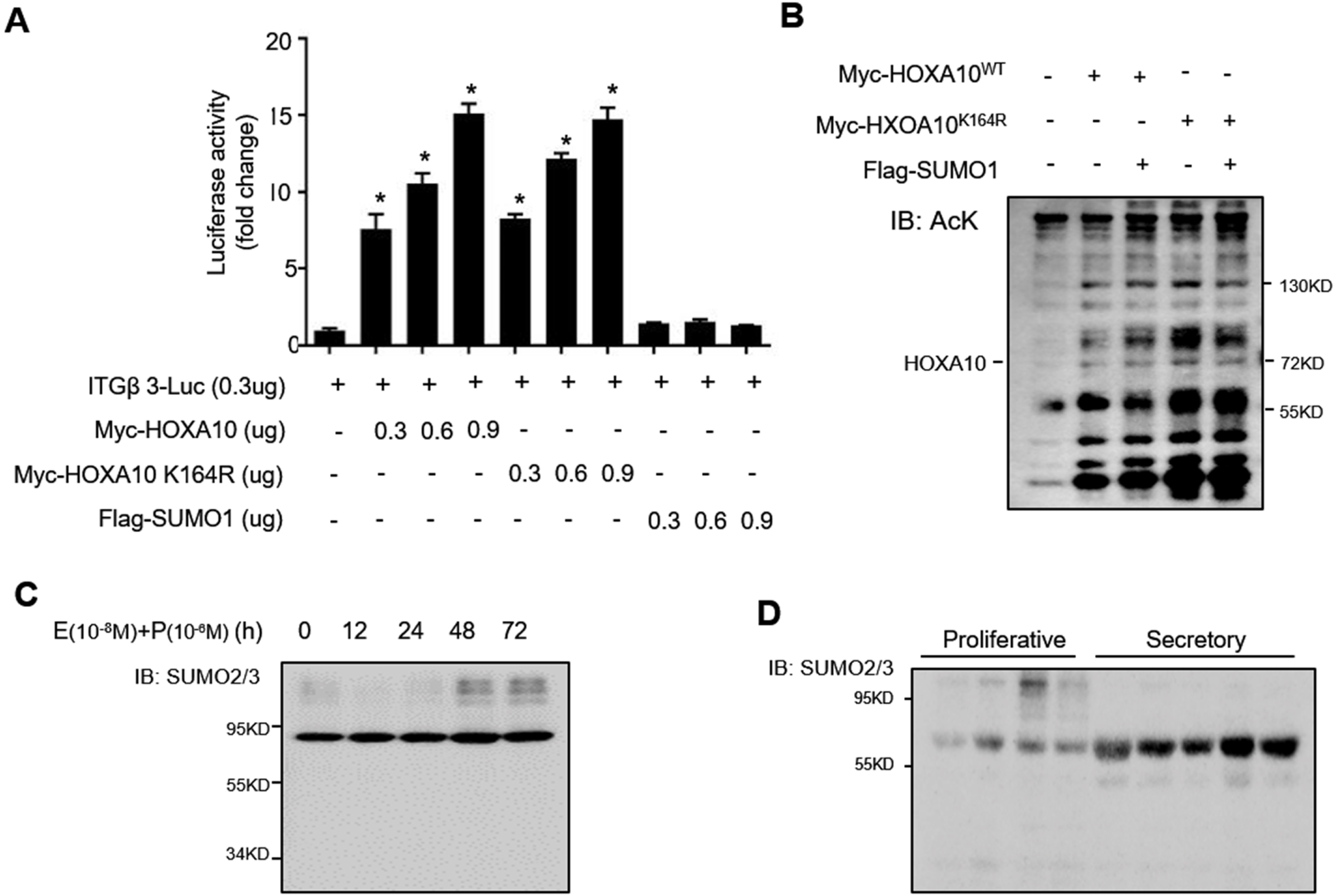

A
Luciferase activity (fold change)
20
15
10
5
0
ITGβ 3-Luc (0.3ug)
Myc-HOXA10 (ug)
Myc-HOXA10 K164R (ug)
Flag-SUMO1 (ug)
0.3 0.6 0.9
B
Myc-HOXA10WT
Myc-HXOA10K164R
Flag-SUMO1
IB: AcK
HOXA10
130KD
72KD
55KD
C
E(10-8M)+P(10-6M) (h)
0 12 24 48 72
IB: SUMO2/3
95KD
55KD
34KD
D
Proliferative
Secretory
IB: SUMO2/3
95KD
55KD

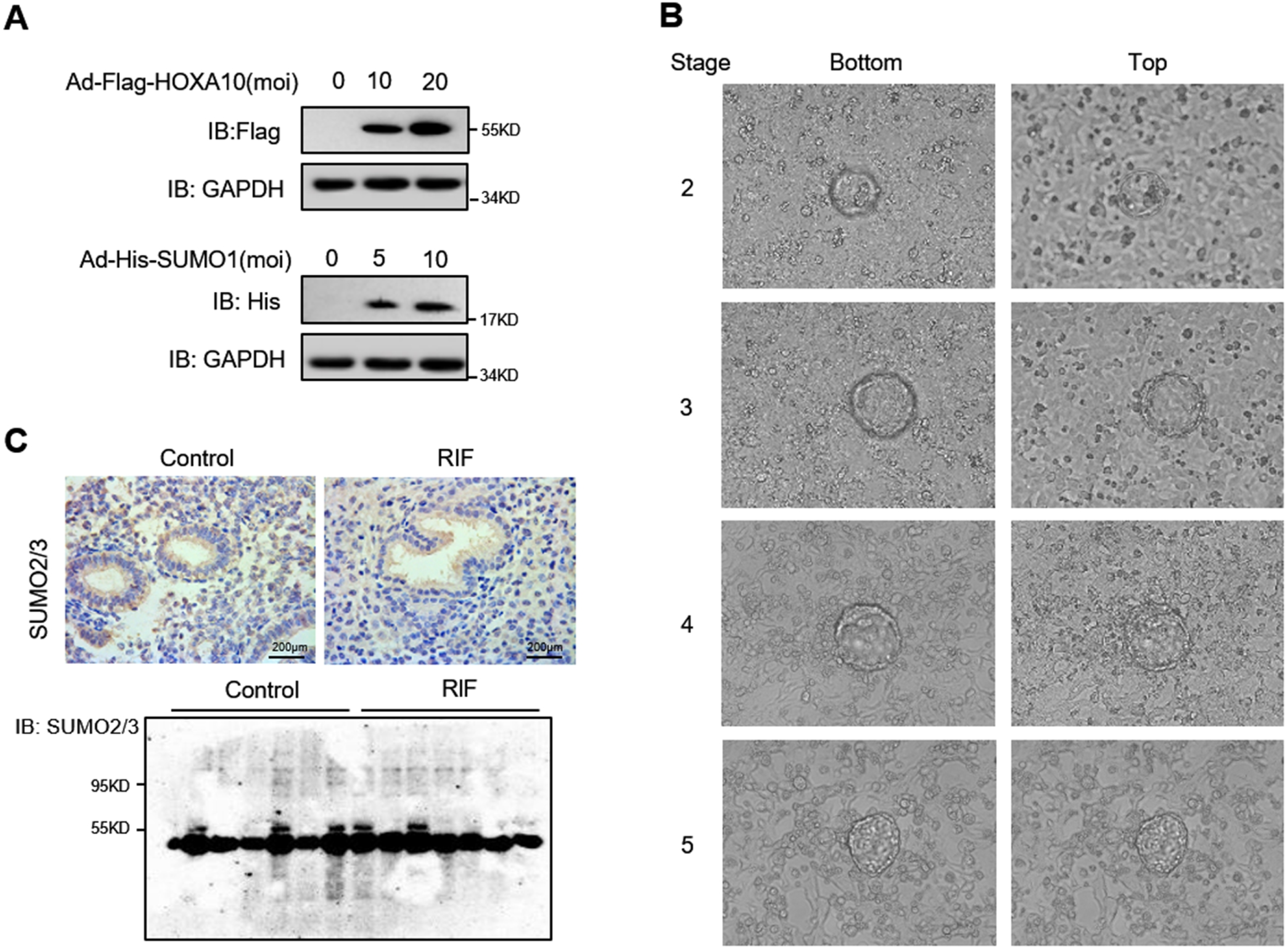

A
Ad-Flag-HOXA10(moi)
0
10
20
IB:Flag
55KD
IB: GAPDH
34KD
Ad-His-SUMO1(moi)
0
5
10
IB: His
17KD
IB: GAPDH
34KD
B
Stage
Bottom
Top
2
3
4
5
C
Control
RIF
SUMO2/3
200μm
200μm
Control
RIF
IB: SUMO2/3
95KD
55KD
